# Bloodborne Viral Hepatitis Infections among Drug Users: The Role of Vaccination

**DOI:** 10.3390/ijerph6010400

**Published:** 2009-01-22

**Authors:** Fabio Lugoboni, Gianluca Quaglio, Paolo Civitelli, Paolo Mezzelani

**Affiliations:** 1 Medical Unit for Addictive Disorders, Department of Internal Medicine, Policlinico GB Rossi, 37134 Verona, Italy; E-Mails: gianluca.quaglio@azosp.vr.it; paolo.mezzelani@univr.it; 2 Addiction Treatment Clinic (SerT-Asl 4), Thiene (Vicenza), Italy; E-Mail: pcivitelli@ulss4.ven.it

**Keywords:** Drug users, hepatitis vaccination, immunization, HBV, HAV

## Abstract

Drug use is a prevalent world-wide phenomenon and hepatitis virus infections are traditionally a major health problem among drug users (DUs). HBV and HCV, and to a lesser extent HAV, are easily transmitted through exposure to infected blood and body fluids. Viral hepatitis is not inevitable for DUs. Licensed vaccines are available for hepatitis A and hepatitis B. The purpose of this overview is to show some epidemiological data about HBV and the other blood-borne viral hepatitis among DUs and to summarize and discuss use of hepatitis vaccinations in this population. Successful vaccination campaigns among DUs are feasible and well described. We try to focus on the most significant results achieved in successful vaccination programs as reported in scientific literature. Vaccination campaigns among DUs represent a highly effective form of health education and they are cost-saving.

## 1. Introduction

Substance abuse and consequent infections are among the most significant problems in the world. All drug users (DUs), not only injecting drug users (IDUs), are at increased risk for infection with at least four different hepatitis viruses: hepatitis A virus (HAV), hepatitis B virus (HBV), hepatitis C virus (HCV), and hepatitis D virus (HDV) [1, 2].

HCV infection is the most common chronic blood-borne infection. An estimated 170 million people throughout the world are infected with HCV, with an estimated 5 million in Western Europe and 4 million in the U.S [3]. Injection of illicit drug is the most important risk factor for HCV infection in developed countries, with prevalence rates as high as 60% to 100%. Hepatitis C virus infection represents the leading cause of chronic liver disease in developed countries. The fall in the deaths from AIDS enhances the problem to prevent and treat HCV infection among DUs [3–10]. HCV can be easily transmitted through exposure to infected blood, far less so through other bodily fluids. The sharing of drug preparation and injection equipment can lead to rapid transmission of HCV among IDUs. Over the last two decades, there have been large increases in the numbers of non-injecting users of heroin in several countries. The sharing of equipment for intranasal use of cocaine and of pipes for smoking crack cocaine may also lead to transmission of these viruses [11–13]. Since there is no vaccine and no postexposure prophylaxis for HCV infection, its management must focus on providing primary prevention efforts.

Hepatitis A is an acute, self-limiting infection of the liver caused by HAV. HAV infection occurs throughout the world, affecting 1.5 million people annually [14]. Oral-fecal is the most common mode of transmission, with parenteral, sexual (specially by anal intercourse) and vertical transmission occurring less frequently. HAV infection is highly correlated with poor water sanitation facilities. Outbreaks have been reported among IDUs for many years and have occurred in the U.S. and Europe. Poor standards of living and unsafe sexual behaviors are strong risk factors leading to transmission. Needle sharing and blood-to-blood transmission have been suggested as possible patterns of transmission among IDUs [14–19].

The World Health Organization has stated that in developed countries hepatitis A vaccination should be considered for DUs. Hepatitis A superimposed on chronic liver diseases is associated with more severe disease, including fulminant hepatic failure and a higher fatality rate [20–23].

HDV is a defective RNA virus that requires the presence of hepatitis B surface antigen (HBs-Ag) from HBV for transmission. In Europe and in the U.S., HDV infection has virtually disappeared, remaining confined to IDUs [33, 34]. IDUs are currently the largest source of HDV infection in the Western world [24–26].

HDV can be acquired either by co-infection with HBV or by superinfection of chronic HBs-Ag carriers. HDV coinfection can cause fulminant hepatitis more frequently than HBV infection alone while HDV supeinfection involves mostly serious chronic liver disease. Because HDV replication requires coinfection with HBV, immunoprophylaxis for HDV infection can be successfully achieved by vaccination against HBV. However, because no effective vaccine specific for HDV has been developed, there is currently no vaccine to protect carriers of HBs-Ag against superinfection.

HBV is carried in blood and in other body fluids and can be transmitted through parenteral and sexual exposure. HBV carriers may have acute hepatitis B or chronic infection. HBV, as well as HCV, may be easily transmitted, not only through sharing needles, but also trough paraphernalia used in the preparation of the drugs. The sexual route is a very common source of infection. HBV is transmitted also perinatally. In the U.S., approximately 1.2 million persons have chronic HBV infection [1–3]. DUs present a higher rate of fulminant HBV hepatitis probably related to the exposure to factors potentiating hepatic damage like alcohol or to HDV coinfection [27].

In Europe, from 20% to 60% of IDUs are seropositive for HBV. The prevalence of HBV infection among DUs attending a methadone maintenance treatment (MMT) in Geneva was much greater among older DUs (80.5%) than younger ones (20.1%) [5, 28, 29]. The seroconversion rate for HBV in a cohort of IDUs enrolled in a syringe exchange program in Malmo was 11.7% per year (compare with no seroconversion among those with HIV) [30].

In the U.S., it is estimated that IDUs comprise 17% of all the new cases of hepatitis B. Among younger IDUs, HBV seroprevalence is about 25%, while for adult IDUs it is often more than 80%, compared with about 5% in the general U.S. population [31]. In 1999, after more than a decade of decline, the incidence of hepatitis B among men aged over 19 and among women aged 40 or over, has increased in the U.S. The most common risk factors among adults continue to be multiple sex partners, male with male sex, and injection drug use [32]; DUs never-injectors infected with HBV and HIV appear to have become infected mainly through sexual transmission, whereas former injectors appear to have become infected with HCV and HIV mainly through injection risk and with HBV through both injection and sexual risk [33, 34]; also among DUs from Rio de Janeiro (BR), HBV seems to be more closely associated with unsafe sex, whereas HCV is positively correlated with high risk injecting behaviour [35]. Prevalence for HBV has high in a large cohort of Italian DUs with no positive tests for syphilis in six years of follow-up [36].

High HBV prevalence among IDUs has been reported in several other countries with low endemic levels for HBV [37–39] similar to prevalence tested in IDUs living in countries highly endemic for HBV [36, 40, 41].

A comprehensive and systematic review targeting HBV and other licensed vaccines immunogenicity in DUs has recently been published [42]. In this paper we have tried to focus on reports that can give concrete help and strategies for health-care providers operating in Addiction Treatment Clinics (ATC) to maximize adherence to hepatitis vaccination campaigns.

## 2. Hepatitis Vaccines

### 2.1. Hepatitis B Vaccine

A vaccine against HBV was developed in 1982, and the first official recommendations for its use in high risk populations were published the same year [31]. For these reasons the HBV vaccine has been the most studied of all vaccines in DUs. Nevertheless, only 10–25 percent of IDUs had isolated antibodies to hepatitis B surface antigen, consistent with vaccine-mediated immunity in U.S. and Europe, and outbreaks of hepatitis B among this population continue to occur [43–45]. Very recent studies evaluating the effectiveness of selective immunization policies in the U.S., U.K. and Spain underlined that vaccination levels remain low in the DU population [46–48]. Despite these discouraging data, good adherence to HBV vaccine has been well documented in North-Eastern Italy [49–53]; the best adherence to vaccination schedule was obtained when vaccine administration was performed directly by addiction treatment clinic (ATC) personnel [51–54]. ATCs that vaccinated less were also those who had worse adherence in vaccination programs [51, 52].

HBV vaccination campaigns targeting IDUs are cost-saving [55]. The most cost-saving strategies include giving the first dose to everyone at screening, administering the vaccination under the accelerated schedule (0, 1, 2 months) and obtaining free or highly discounted vaccine from local health departments; to offer HBV screening in the absence of vaccination has been proved economically inappropriate [55].

We cannot omit the possible impact of universal vaccination on DUs; according to WHO recommendations, universal vaccination has been currently implemented in 168 countries worldwide with an outstanding record of safety and efficacy. In Italy, vaccination against hepatitis B became mandatory for all newborns and adolescents starting from 1991, first in the world; thus, it’s of crucial importance to asses whether vaccinated subjects are still protected during adulthood when risk of infection with HBV for lifestyle can increase. A long-term immunogenicity after universal vaccination has been found in a large cohort of Italian healthy adolescents 10 years after vaccination. Universal vaccination has shown a great impact in the control of hepatitis in a former hyperendemic area in South Italy, but data regarding DUs is actually lacking [56–58]. Although universal vaccination could change the spread of HBV also among DUs, two major issues will remain: the real effectiveness of newborn vaccination and infection control among DUs coming from high endemicity countries.

Suboptimal immunological responses (58%–77%) have been reported among DUs despite the 95–99% proven in young adults from the general population [42, 59]. Dysfunction of cell-mediated immunity, alcohol use, polydrug abuse, multiple bacterial infections, HCV positivity and malnutrition are possible explanations of the lower immune response to anti-HBV vaccination in IDUs. More recently, smoking status has been significantly related with lower antibody response to HBV vaccine in patients with chronic HCV [60]. This observation is relevant because tobacco smoking and HCV infection are extremely prevalent among DUs and methadone maintenance patients but, despite this fact, substance abuse treatment programs too often ignore tobacco use [61]. Self-reported HBV infection status and immunization status in DUs have proved to be unreliable and the 52% claiming to be vaccinated were tested susceptible to HBV. As a result, some have suggested a “Don’t ask, vaccinate” policy for DUs [62].

Carriers of HCV chronic hepatitis normally respond worse than HCV negative subjects to HBV vaccination, and this negative influence seems to be related to HCV viral load [51, 63, 64]. In HCV positive subjects a larger dosage of vaccine antigen or multiple booster doses may be useful [65]. Substained viral response following interferon plus ribavirin treatment does not affect the antibody response to HBV vaccine, and vaccination during antiviral therapy is not beneficial in terms of anti-HBs seroconversion rates [66]. HBV vaccination proved safe in chronic HCV patients. Several studies showed that an immunological response to HBV vaccination in patients infected with HIV was lower than in the normal population [42, 51, 59, 67], but a recent study has documented good seroconversion rates to HBV vaccine in patients with AIDS and virological response to highly active antiretroviral therapy (HAART) [68]. There is good evidence that individuals without detectable anti-Hbs levels for many years still have strong anamnestic immune responses upon in-vitro stimulation [42]. So far only one prospective cohort study has evaluated the long-tern benefits of vaccination in DUs [69].

Vaccination among IDUs have been reported to be well tolerated, similar to the general population [51]. Pre-immunization testing may be cost effective among IDUs, where the expected prevalence of prior infection exceeds 30% [59, 67]. One study showed an interesting relationship between vaccination for hepatitis B and HCV serostatus, among DUs; in this study vaccinating DUs against HBV may have helped to create a stronger pro-health attitude leading to a reduction in HCV risk behaviour [7]. The presence of the antibody to hepatitis B core antigen (anti-HBc) in the absence of other HBV markers is common among DUs [70, 71]. The response to HBV vaccine in isolated anti-HBc carriers appears to vary greatly [42, 51, 63, 72]. Follow-up studies of IDUs with isolated anti-HBc showed resistance to HBV re-infection, indicating that these patients do not require vaccination [70]. High Prevalence of occult HBV in IDUs has been demonstrated [73]. One study had also demonstrated the disappearance of HBV-DNA in a significant proportion of patients HCV-infected with occult HBV infection and anti-HBc-positive blood donors after HBV vaccination; but no data on DUs has been reported [74].

The low coverage for HBV vaccination among DUs may be attributed to a combination of factors: the absence of targeted healthcare programs to provide hepatitis B vaccination for this risk group, the low number of health workers with the required training to carry out vaccination in ATC and the lack of awareness among IDUs about the risk of hepatitis [75–77].

Given the high risk of HBV infection, a lower post-vaccination seroprotection rate, and difficulties in follow-up with DUs, it is important to administer the vaccination whenever possible. Several studies have shown that IDUs miss a number of opportunities for HBV vaccination [78–80]. If vaccination were available at many more kinds of sites, many more IDUs could receive vaccination. For example, methadone maintenance clinics and other drug treatment programs that require frequent attendance could provide vaccination services. Several studies have demonstrated a very good adherence to vaccine with high completion rate in these facilities [49–54]. HBV vaccine also can be successfully offered in on-site needle exchange services [78–80]. CDC recommends HBV vaccination in correctional facilities, where a high percentage of DUs are present [81]. Other venues that could provide immunization are emergency departments [82].

The goal should be to offer HBV vaccination as soon as possible after the start of illicit drug use and to achieve the highest possible rate of complete vaccination coverage. The inability to ensure high rates of completion of vaccine schedule should not preclude the initiation of vaccination. Protective levels of antibody develop in 30% to 55% of adults following a single dose of vaccine and in 75% after 2 doses; therefore, a percentage of DUs who have not completed the vaccination series are probably protected against HBV infection, even if at a lower rate than above reported [50]; no harm has been documented for receiving more than three doses. Because most hepatitis infections are asymptomatic, greater efforts should be made to increase the access to screening, but HBV screening in the absence of vaccination is not cost-effective [55, 79].

### 2.2. Hepatitis A Vaccine

Even if incidence of HAV has generally decreased in many parts of the world with improved hygienic conditions, many recent epidemics have been described among DUs. Approximately 40 to 50% of IDUs in northern Europe are anti-HAV positive; but it should be noted that, in some areas, the HAV prevalence among IDUs does not differ from that of the general population. Cross-sectional serologic surveys have shown IDUs to have higher anti-HAV seroprevalence than the general U.S. population [83–85]. Subjects with HAV infection are viremic for as long as 30 days before the onset of symptoms suggesting that the opportunity for parenteral transmission may be greater than previously suspected [86].

Since 1995, there have been two effective HAV vaccines on the market and the WHO has recommended HAV vaccination for DUs [29, 67]. Vaccination is recommended also in order to prevent superinfection in HCV positive subjects, which can be severe.

A two-dose schedule is recommended for HAV vaccine, with the second dose 6–18 months after the first. Inactivated hepatitis A virus vaccines are highly immunogenic in the general population. Neutralizing antibodies are present in more than 95% of the vaccinated population one month after the first dose and almost all recipients have a response after the second dose [84, 87]. Similar to experimental observations with HBV vaccination, DUs respond weakly to vaccination compared with the general population; the only two studies that analyzed seroconversion among DUs reported a good immunogenicity only after the second dose [22, 88]. Comparable results have emerged in subjects with chronic liver disease [89]. The timing of the booster dose is not critical in healthy subjects, but in DUs the second dose is quite crucial and must be administered after a shorter time than after 6–18 month as normally scheduled, in order to reduce the unprotected period [31, 90].

A lower geometric mean titer (GMT) could affect the duration of protection. There is no evidence, unlike with the immune response to HBV vaccine, that individuals formerly seroconverted but without detectable anti-HAV levels still have immune protection against HAV. However, single-dose immunization targeting DUs has been demonstrated to give enough protection to end HAV outbreaks in different settings (42). The persistence of anti-HAV titers, the duration of protection, and the possible need for additional vaccine booster doses in different schedules should be studied in future research.

### 2.3. Hepatitis A–Hepatitis B Combined Vaccine

Since 1996, in addition to the monovalent vaccines against hepatitis A and B, a combined vaccine containing HBs-Ag and HAV-Ag has been available. This vaccine, given in a three-dose schedule at 0, 1, and 6 months, appears to be both as safe and as effective as monovalent vaccines [91].

Studied for the first time in DUs, the combined vaccine proved to be safe and immunogenic [53]. The seroconversion rate was comparable with that for the general population; however, GMT for HAV and HBV, as documented with the other vaccines among DUs, proved lower than those reported in the literature. One interesting item emerging from this study was that the anti-HBV and anti-HAV responses were much higher than in the case of monovalent vaccines in two cohorts of DUs. It is possible that the combined vaccine could have an adjuvant effect for both components (mutual immune reinforcement), as reported for the general population. This effect would be particularly useful in DUs because the peak antibody titre is relevant in providing a longer protection, specially for HAV. Even if further studies are required, this study suggests that IDUs who are HAV/HBV-negative could be better vaccinated with the combined vaccine than with monovalent ones [22, 50, 53].

## 3. Discussion

All of Authors of this paper have been working as staff members in vaccination campaigns among DUs. We have tried to point out, through summarizing published data and, also, from our own experience, the essential take-home messages for safe, successful vaccination programs in hard-to-reach populations.

Remember that:

- Good addiction therapy means good adherence to vaccination programs. Multi-disciplinary management models have proved to be effective, among DUs, in vaccinating and in treating HCV chronic hepatitis as well [92].
- Vaccination programs with minor drop-out turn out to be those administrated directly by ATC personnel. In our experience this is the first of all issues.
- ATCs which vaccinate less are also those that have worse adherence in vaccination programs.
- HBV screening in the absence of free access to vaccination is a wasted opportunity.
- HBV vaccine boosters are no longer made to patients who have documentated seroconversion even if anti-HBs titre get lost. This is not true for HAV vaccine.
- Main issue is no longer to quantify anti-HBs titre, but if there is any seroconversion or not. On that purpose it is important to follow the vaccine schedule (0, 1, 6 months or 0, 1, 2 months) with a serum control at the end; a booster dose in lack of response can be determinant. No harm has been reported from receiving more than three doses.
- Short schedule (0, 1, 2 months) reduces drop-out; it’s cost-saving even if it is proved to be less immunogenic comparing with the usual one (0, 1, 6 months).
- DUs are less responsive to hepatitis vaccines than the general population both in immunogenicity and reactogenicity.
- IDUs with isolated anti-HBc they showed resistance to HBV re-infection in follow-up studies and, in our opinion, these patients do not require HBV vaccination.
- Self-reported HBV infection status and immunization status in DUs have proven to be unreliable. However, even if some clinicians adopt a “Don’t ask, vaccinate” policy, we believe that good data collecting can be easier, less expensive and more effective among DUs in methadone/buprenorphine maintenance programs.
- It should be remembered that HIV positive subjects seroconverted to anti-HBs less than HIV negative but also that patients with AIDS and virological response to HAART have a good immunological response to HBV vaccination.
- Carriers of HCV chronic hepatitis normally respond worse than HCV negative to HBV vaccination; they deserve a more careful follow-up.
- Scheduling for HAV vaccination has been designed mainly for travelers who need fast protection; when evaluated among DUs it proved to be far less immunogenic and reactogenic than in the general population. Our opinion is that a booster dose is needed soon after the first dose.
- A combined vaccine (HAV and HBV) can give better results than a monovalent one both in immunogenicity and seroconversion rate. This fact could be relevant for a longer protection, specially against HAV.
- Vaccinating DUs against hepatitis may help to create a stronger pro-health attitude leading to a reduction in HCV and HIV risk behavior.
- Preventing HBV and HAV among DUs can really limit the spread in the general population and it has been demonstrated to be cost-saving.

## 4. Conclusions

The literature briefly reviewed in this paper suggests that substance abuse and blood-borne hepatitis are highly prevalent in the world today, with serious morbidity and mortality complications. DUs are at high risk for vaccine-preventable infections but generally have the lowest vaccination coverage rates. The health consequences extend beyond individual DUs to their sexual partners, households and communities as a whole, until universal vaccination reaches the high-risk age. Successful vaccination campaigns among DUs have been demonstrated to be feasible and effective.

Further studies are required to clarify situations correlated with a lower immune response to vaccinations in DUs and for tailored vaccination schedules giving better immune responses. Data on HAV vaccination are still scarce. The ability of DUs to improve their health and reduce the risk of acquiring or transmitting hepatitis is directly related to the quality of the prevention and care services they receive. In future we hope that vaccines for HIV and HCV will be developed. It would be paradoxical indeed if, at some time, coverage of DUs will be stopped for lack of experience in performing vaccine programs among this vulnerable group.

Improving HAV and HBV vaccination programs for DUs represents a highly effective and cost-saving form of health action.

### Conflicts of Interests

We declare that we have no conflict of interest.
